# The Role of T2 Mapping in Cardiac Amyloidosis

**DOI:** 10.3390/diagnostics14101048

**Published:** 2024-05-18

**Authors:** Giulia Grazzini, Silvia Pradella, Rossella Bani, Chiara Fornaciari, Francesco Cappelli, Federico Perfetto, Diletta Cozzi, Simona Giovannelli, Giacomo Sica, Vittorio Miele

**Affiliations:** 1 Department of Radiology, Careggi University Hospital, Largo Brambilla 3, 50134 Florence, Italy; pradella3@gmail.com (S.P.); bani.rossella@gmail.com (R.B.); chiarafornaciari2@gmail.com (C.F.); dilettacozzi@gmail.com (D.C.); simona.giovannelli@unifi.it (S.G.); vmiele@sirm.org (V.M.); 2 Regional Amyloid Center, Azienda Ospedaliero Universitaria Careggi, Largo Piero Palagi 1, 50134 Florence, Italy; f.cappelli@unifi.it (F.C.); federico.perfetto@unifi.it (F.P.); 3 Department of Radiology, Monaldi Hospital, Azienda Ospedaliera dei Colli, 80131 Naples, Italy; gsica@sirm.org

**Keywords:** cardiac amyloidosis, cardiac magnetic resonance, T2 mapping

## Abstract

Cardiac amyloidosis (CA) is an infiltrative cardiomyopathy divided into two types: light-chain (LA) and transthyretin (ATTR) CA. Cardiac magnetic resonance (CMR) has emerged as an important diagnostic tool in CA. While late gadolinium enhancement (LGE), T1 mapping and extracellular volume (ECV) have a consolidate role in the assessment of CA, T2 mapping has been less often evaluated. We aimed to test the value of T2 mapping in the evaluation of CA. This study recruited 70 patients with CA (51 ATTR, 19 AL). All the subjects underwent 1.5 T CMR with T1 and T2 mapping and cine and LGE imaging. Their QALE scores were evaluated. The myocardial T2 values were significantly (*p* < 0.001) increased in both types of CA compared to the controls. In the AL-CA group, increased T2 values were associated with a higher QALE score. The myocardial native T1 values and ECV were significantly (*p* < 0.001) higher in the CA patients than in the healthy subjects. Left ventricular (LV) mass, QALE score and ECV were higher in ATTR amyloidosis compared with AL amyloidosis, while the LV ejection fraction was lower (*p* < 0.001). These results support the concept of the presence of myocardial edema in CA. Therefore, a CMR evaluation including not only myocardial T1 imaging but also myocardial T2 imaging allows for more comprehensive tissue characterization in CA.

## 1. Introduction

Cardiac amyloidosis (CA) is a rare, progressive disease characterized by extracellular amyloid deposition, leading to severe cardiac dysfunction and death. Over 98% of CA originates from either misfolded monoclonal light-chain (AL) proteins or from transthyretin (ATTR), a protein tetramer released by the liver that carries circulating thyroxine- and retinol-binding proteins, which can occur in both the acquired (ATTRwt, wild-type) and dominantly inherited forms (ATTRv, variant) [1]. It is well known that amyloid fibrils may induce cardiac damage by dual mechanisms: (1) the continuous widespread deposition of amyloid fibrils along every cardiac structure, with progressive disruption of the myocardial tissue architecture, and (2) through the toxic effects exerted by prefibrillar oligomers, particularly in AL amyloidosis, which compromise cellular function and induce organ dysfunction [2,3,4].

Early diagnosis of cardiac amyloidosis is crucial, mainly because the overall survival is poor once overt cardiac involvement is present. Diagnosis of CA requires a multimodality approach employing echocardiography, cardiac magnetic resonance (CMR) and nuclear imaging. With detailed tissue characterization, high-resolution imaging and robust cardiac assessment, CMR enables a comprehensive investigation of CA using parameters both visual and quantitative. Moreover, CMR has emerged as a tool for identifying early disease and evaluating disease progression and response to therapy [5,6,7].

In CMR imaging, late gadolinium enhancement (LGE) sequences, T1/T2 mapping and extracellular volume (ECV) measurement provide useful insights for diagnosis, patient stratification and prognostication [8]. The LGE pattern highly suggestive of CA is a diffuse or circumferential subendocardial or transmural enhancement, even if other LGE patterns have also been described [9]. T1/T2 mapping and ECV allow for the quantification of diffuse myocardial tissue changes, such as amyloid infiltration [10,11]. Myocardial native T1 mapping and measurement of ECV showed great potential in CA stratification and evaluation of the disease’s response to therapy. CA is associated with increased myocardial native T1 and ECV values [12]. Fontana et al. found AL amyloidosis showed higher myocardial native T1 values compared to ATTR amyloidosis [13]. In AL-CA patients, higher native T1 values were associated with a worse prognosis [14]. The quantification of ECV, with the addition of contrast, can potentially provide insight into the degree of amyloid infiltration, as amyloid fibril deposition leads to the gradual expansion of the extracellular matrix [15]. Moreover, ECV can be an early marker of disease, as it increases before LGE [16]. More importantly, ECV has shown important prognostic utility in both AL- and ATTR-CA, as it decreases in patients with a complete or partial response to therapy [16]. In contrast to T1 mapping and ECV, T2 mapping has been less often evaluated in CA, and conflicting results have been reported. However, several studies have suggested that amyloid infiltration is not the only mechanism of myocardial injury in cardiac amyloidosis and that myocardial edema could be an important component in this picture [17,18].

Myocardial edema is defined as intra- or extracellular water accumulation [19]. It increases usually in acute myocarditis and in acute myocardial ischemia [20,21]. However, there are also noninflammatory mechanisms leading to myocardial edema, such as increased afterload [22]. CMR can detect myocardial edema in vivo through the visualization of a high signal intensity in T2-weighted edema-sensitive images and, even better, through the quantification of elevated T1 and T2 values in T1/T2 mapping sequences. The T2 relaxation time is a CMR biomarker more specific to the free water content in tissue compared to that in T1 [23]. In fact, the T1 relaxation time is influenced by both extracellular and intracellular compartments, so both free water and the water bound to large molecules such as amyloid may increase the T1 values [24,25].

In this setting, we aimed to test the additive value of T2 mapping in the evaluation of cardiac amyloidosis through a comprehensive CMR evaluation.

## 2. Materials and Methods

### 2.1. Study Population

Patients with amyloidosis recruited to our Regional Amyloid Center underwent comprehensive assessment, including clinical evaluation, echocardiography and serum and urine biochemistry, comprising N-terminal pro-b-type natriuretic peptide, serum creatinine and troponin-T. In addition, the CA patients underwent 1.5 T CMR. A diagnosis of ATTR-CA was made using the established non-invasive criteria according to the latest European Society of Cardiology guidelines and confirmed by endomyocardial biopsy when needed [26]. AL was diagnosed in the presence of a monoclonal component and confirmed by tissue biopsy with amyloid subtyping of the involved organ or fat pad. All patients with ATTR-CA underwent genetic testing.

We retrospectively evaluated all the CMR images of the CA patients obtained from April 2012 to April 2023. The exclusion criteria were poor image quality, the presence of artefacts in the LGE-CMR and T2 mapping sequences and incomplete CMR examination (due to claustrophobia or the absence of T2 mapping sequences). A total of 70 subjects were finally included in the present study and were categorized into two groups: AL-CA and ATTR-CA. A control group of 30 healthy subjects (18 males; 42 ± 12 years) was also included in the study and explored with CMR.

This retrospective single-center study was conducted in accordance with the Declaration of Helsinki’s Ethical Principles and Good Clinical Practices and was approved by the Biomedical Research Ethics Committee of our institution. All patients gave their informed consent to the CMR examination with intravenous administration of the contrast media. The patients’ records were anonymized prior to analysis.

### 2.2. CMR Protocol

The CMR images were acquired by employing three 1.5 T scanners (Philips Ingenia, Philips, Best, the Netherlands; MAGNETOM Sola, Siemens Healthineers, Erlangen, Germany; MAGNETOM Aera, Siemens Healthineers, Erlangen, Germany). Mapping reference values for each scanner were defined by collecting myocardial T1 and T2 values from 30 healthy subjects. The reference ranges are summarized in Table 1.

Breath-hold, retrospectively ECG-gated, cine, steady-state, free processing images were acquired in the short axis (SA), covering the entire left ventricular (LV) (6 mm slices without a gap), and in the 2-chamber (along a plane passing between the center of the mitral–tricuspid annulus and the apex of the left and right ventricles), 3-chamber (along a plane passing between the aortic and mitral annulus and the apex of the left ventricle) and 4-chamber long-axis views (along an image plane passing through the center of the left ventricular cavity and the right ventricular costophrenic angle).

Short Tau inversion recovery (STIR) T2-weighted images were acquired in the middle short-axis section to assess myocardium edema. The T2 maps were performed using a three-slice ECG-triggered multi-echo turbo spin echo, combined with a black blood pre-pulse to create the T2 map images. The sequences performed were T2-prepared gradient echo (T2p-GRE) or gradient spin echo (GraSE), according to the scanner type.

T1 mapping was performed using ECG-triggered Modified Look–Locker Inversion recovery (MOLLI) acquisitions, with the scheme 5(3)3. Post-contrast T1 mapping was performed using the same sequence and slice positions, with the scheme 4(3)2.

The SA LGE-CMR images were acquired using breath-hold, ECG-triggered inversion recovery gradient echo sequences ten minutes after the administration of 0.15 mmol/kg of Gadobutrol (Gadovist, Bayer Healthcare), infused at 1 mL/s, followed by 20 mL of saline with an automated injector. The inversion time was calculated for each patient by considering the null point of the normal myocardium signal using a Look–Locker sequence.

All the scan parameters are summarized in Table 2.

### 2.3. Image Analysis

The post-processing analysis was performed using dedicated software (Cvi-42 Circle Cardiovascular Imaging^®^, Cvi-42 version 5.13, Calgary, AB, Canada). Endocardial contours were automatically drawn during both the diastolic and systolic phases to evaluate the LV volumes and systolic function. Epicardial contours were defined in the diastolic phase to evaluate the LV myocardial mass. Volumes were collected as absolute and indexed values. Ventricular volumes, ejection fraction and myocardial mass were obtained from post-processing analysis on 5–10 mm cine-SA-CMR slices, covering the LV and right ventricular (RV) and acquired in short view.

**Figure 1:**
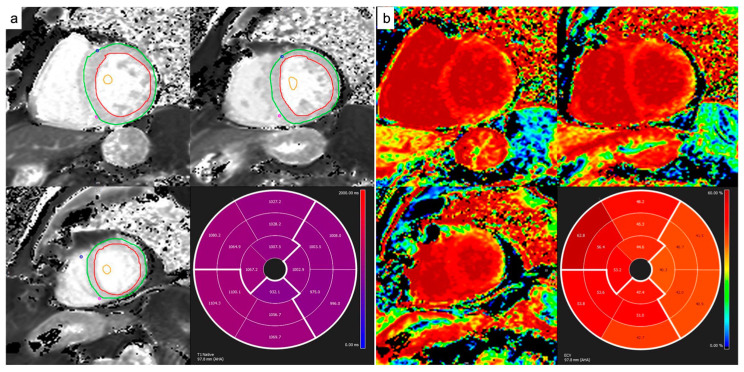
Native T1 and ECV calculation from MOLLI sequences. Endocardial and epicardial contours and blood pool ROIs were drawn on pre-contrast and post-contrast maps in order to quantify native T1 (**a**) and ECV values (**b**), respectively.

The myocardial native and post-contrast T1 relaxation images were first manually segmented, drawing endocardial and epicardial contours, and then were co-registered to eliminate motion-related artifacts using Cvi42 software. Subsequently, the automatically derived global T1 and ECV maps were visually checked for the presence of artifacts. The T1 values of the blood pool before and after contrast administration were calculated from a region of interest (ROI) placed in the blood pool cavity in the middle short-axis section (Figure 1).

**Figure 2:**
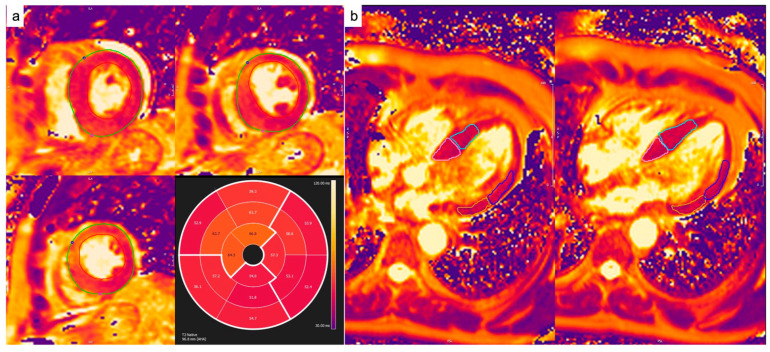
Native T2 calculation from T2p-GRE or GraSE sequences. Endocardial and epicardial contours were drawn on short-axis pre-contrast maps (**a**). On four-chamber images (**b**), four different ROIs per slice were drawn in order to quantify native T2 values at the basal and mid septa and at the basal and mid lateral walls.

In order to establish the presence of edema in the T2w sequences, a qualitative analysis through visual estimation was performed. For quantitative analysis of the native T2 values, the short-axis or 4-chamber images were manually contoured to outline the endocardium and epicardium, and the T2 values were calculated using dedicated software (Cvi42) (Figure 2).

In order to calculate the mean native T1, T2 and ECV values, we considered only the septa and lateral and anterior walls at the base and mid-ventricle. In our analysis, we did not include the posterior wall or apical segments due to their sensitivity to artifacts.

The segmentation process for T1 and T2 mapping was not time-consuming thanks to the radiologists’ experience (at least 10 years) and the automatic software steps.

Myocardial enhancement was also evaluated and was quantified using the Query Amyloid Late Enhancement (QALE) score. The QALE score was performed on LGE images at the base, mid-ventricle and at the apex in the LV and RV. Each LV level is scored according to the degree of LGE, with the highest score for circumferential and transmural LV-LGE. The QALE score range for the whole heart in each patient was from 0 (no detectable LGE in the LV or RV) to 18 (global transmural LV-LGE at all 3 levels, plus RV involvement). Abnormal enhancement of the atria was also noted.

### 2.4. Statistical Analysis

Statistical analysis was performed with SPSS version 20.0 (IBM Corporation, Armonk, New York). All the continuous variables are presented as means and standard deviation or as medians and interquartile ranges, whereas categorical variables are presented as counts and percentages. Continuous variables were compared using parametric measures (2-sample Student’s t-test, 1-way analysis of variance after checking for homogeneity of variance, with post hoc Bonferroni). Spearman’s rank correlation coefficients were calculated to assess the correlation between continuous variables.

Categorical variables were evaluated using the χ2 test. The correlation between the two scores was tested by means of Pearson’s test.

A two-tailed *p* value < 0.05 was considered statistically significant.

## 3. Results

### 3.1. Population Characteristics

Seventy consecutive CA patients who underwent CMR between April 2012 and April 2023 at our center were retrospectively identified. AL-CA patients constituted 19 (27%) of them, and ATTR-CA patients constituted 51 (73%). Among the ATTR-CA group, 15 (29%) were mutant type and 36 (71%) were wild type.

The patients with ATTR amyloidosis were older compared with the AL amyloidosis patients (mean age 77 versus 71 years; *p* = 0.01) and predominantly male (84% vs. 47%; *p* = 0.02). The ATTR-CA patients had a larger body surface area (Mosteller BSA) than the AL-CA patients (1.89 versus 1.72, *p* = 0.03). No significant differences were detected between the AL-CA and ATTR-CA patients regarding their biological and echocardiographic parameters.

All data are reported in Table 3.

### 3.2. CMR Image Analysis

#### 3.2.1. Qualitative Analysis and Quantitative Analysis

A total of 67 (97%) patients exhibited enhancement of the LV in the LGE images, with 48 (96%) in the ATTR-CA group and 19 (100%) in the AL-CA group. The mean QALE score was significantly higher in the ATTR-CA patients than in the AL-CA patients (10.8 ± 4.0 and 8.5 ± 5.3, respectively; *p* = 0.049). Atrial involvement was found in 47 patients (68%), with 37 (74%) in the ATTR-CA group and 10 (53%) in the AL-CA group.

LV mass was significantly (*p* < 0.001) higher in the ATTR-CA than in the AL-CA patients (109.7 ± 37.8 g/m^2^ and 78.9 ± 26.2 g/m^2^, respectively). Compared to the ATTR-CA patients, the AL-CA subjects had a significantly (*p* = 0.003) greater LV ejection fraction (LVEF), while LV stroke volume (LV-SV), LV end-diastolic and end-systolic volume (LV-EDV and LVESV) and interventricular septum thickness (IVS) were higher in the ATTR-CA subjects.

#### 3.2.2. Mapping Analysis

**Figure 3:**
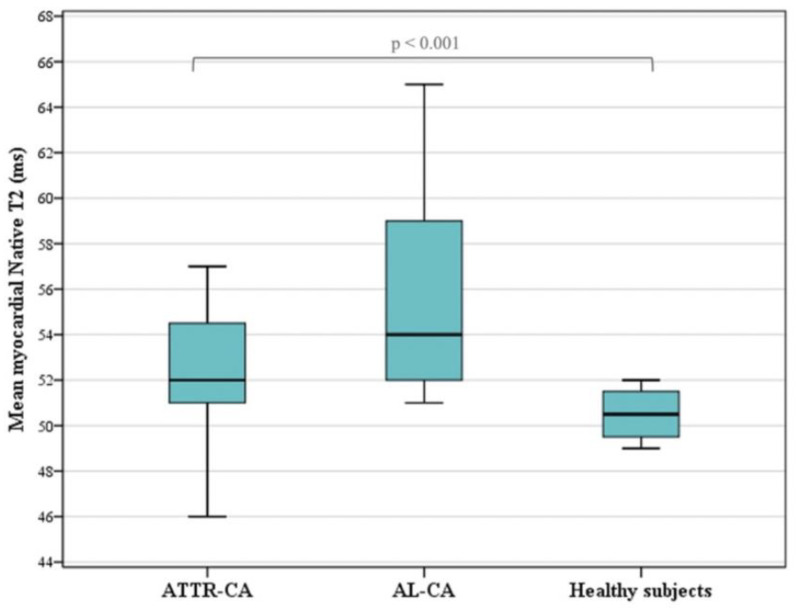
Myocardial native T2 values are significantly (*p* < 0.001) higher in CA patients than in healthy subjects, as shown by the boxplots of T2 values in healthy subjects, patients with AL amyloidosis and patients with ATTR amyloidosis.

The myocardial T2 values were significantly (*p* < 0.001) increased in 54 of the subjects (77%) compared to the controls. The overall mean T2 value was 53.9 ± 4.8 ms. Higher T2 values were found in the AL-CA patients (56.0 ± 5.5 ms) than in the ATTR-CA patients (53.2 ± 4.5 ms) but without reaching statistical significance in our population (*p* = 0.260) (Table 4). The mean native T2 value did not differ by gender (Figure 3).

**Figure 4:**
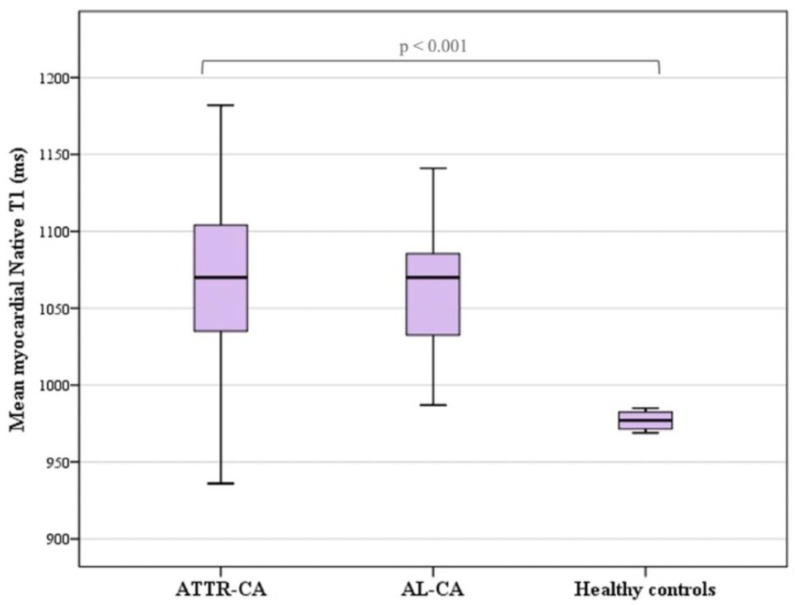
Myocardial native T1 values are significantly (*p* < 0.001) higher in CA patients than in healthy subjects, as shown by the boxplots of T1 values in healthy subjects, patients with AL amyloidosis and patients with ATTR amyloidosis.

A significant rise in the myocardial native T1 values was also noticed in all the CA patients (*p* < 0.001) (Figure 4). The overall mean T1 native value was 1065 ± 55 ms. No differences were found in the native T1 values between the AL-CA patients and the ATTR-CA patients (1066 ± 59 ms and 1062 ± 42 ms, respectively; *p* = 0.774) (Table 4).

**Figure 5:**
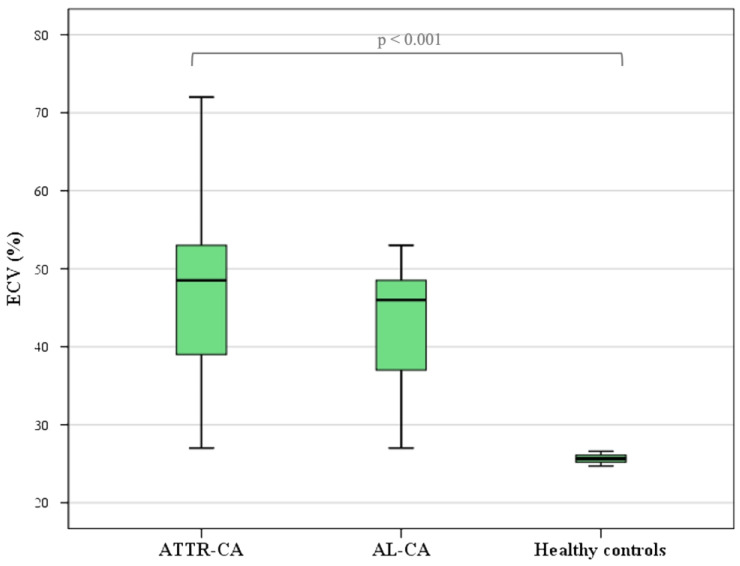
ECV is significantly (*p* < 0.001) higher in CA patients than in healthy subjects, as shown by the boxplots of ECV values in healthy subjects, patients with AL amyloidosis and patients with ATTR amyloidosis.

We found that ECV was strongly increased in 69 patients (99.9%, *p* < 0.001) (Figure 5), with higher values in the ATTR-CA patients than the AL-CA patients (overall mean ECV = 48 ± 11; mean ECV in subgroups was 49 ± 10 in ATTR-CA and 44 ± 12 in AL-CA), without statistical significance in our population (*p* = 0.202) (Table 4).

#### 3.2.3. Relationship between Myocardial Native T2 Values and Other Parameters

In the overall population, there was no correlation between increased native T2 values and markers of disease severity, including native T1 values, ECV, extent of LGE and NT-proBNP, nor a correlation with New York Heart Association functional classes or different grades of diastolic dysfunction. The same results were obtained in the ATTR-CA group.

**Figure 6:**
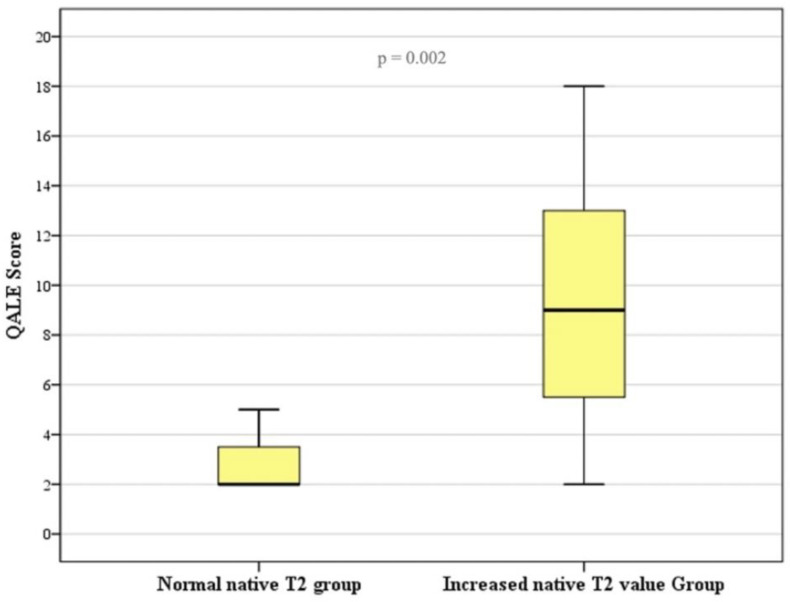
Graph shows QALE score values in AL-CA patients with normal native T2 values and with increased native T2 values. QALE score is significantly (*p* = 0.002) higher in CA patients with increased native T2 values.

In the AL-CA group, increased T2 values were associated with greater LGE extension, expressed as a QALE score value (9.5 ± 5.1 in AL-CA patients with high native T2 values and 3.0 ± 1.7 in the other AL-CA patients; *p* = 0.002) (Figure 6).

All the MRI data are reported in Table 4.

## 4. Discussion

In this study, we reported a significant rise in myocardial native T2, T1 and ECV values in patients with CA compared to the controls. The myocardial T2 values were increased in most of the CA patients, while the T1 values were elevated in all of them. In our series, we did not observe statistically significant differences between the native T2 values of the AL-CA and ATTR-CA patients, even if the T2 values were higher in the AL-CA patients than in the ATTR-CA patients. Furthermore, we found no statistically significant differences in terms of the T1 and ECV values between the AL-CA and ATTR-CA patients, even if the ECV values were lower in the AL-CA than in the ATTR-CA patients.

In amyloidosis, the major determinant of survival is the presence and degree of cardiac involvement [27,28]. CMR has emerged as a tool able to measure amyloid burden, so it is now considered the imaging of choice for the diagnosis of cardiac involvement in systemic amyloidosis [29]. However, a discordance poorly understood has emerged. AL-CA patients generally have a worse prognosis, with the median survival from presentation being 6 months in AL amyloidosis and 6 years in ATTR amyloidosis [30]. Nevertheless, amyloid infiltration is usually more severe in ATTR-CA than in AL-CA, with the LV mass, LGE and ECV higher than in AL-CA [13]. Our study confirmed these data, showing a higher LV mass, QALE score and ECV in the ATTR-CA subjects than in the AL-CA patients, while the LVEF was lower. This discordance suggests that another component in addition to amyloid infiltration is involved in myocardial injury in cardiac amyloidosis. Moreover, AL-CA showed higher myocardial native T1 and lower ECV values compared with ATTR-CA, suggesting that myocardial edema could be the additional component in this picture [17]. Kotecha et al., in their study on 286 patients with cardiac amyloidosis, found the T2 mapping values were higher in patients with untreated AL-CA compared with treated AL-CA and ATTR-CA, and T2 was an independent predictor of prognosis in AL amyloidosis. Moreover, they demonstrated histological evidence of myocardial edema in 87.5% of biopsies, with the extent of edema ranging from 5% to 40% myocardial involvement [18]. Our results are in line with Kotecha et al., showing significantly increased T2 values in 77% of the CA patients. These results suggest that cardiac amyloidosis edema is not always present or alternatively sometimes is so restricted that we cannot perceive it.

For these reasons, recently, there has been increasing interest in performing T2 mapping in cardiac amyloidosis, even if the results are controversial in the literature and more studies are necessary. Sparrow et al. did not find significant differences between CA patients and controls in terms of T2 relaxation time [31]. However, other studies [5,18,32,33] in the literature showed an elevation in T2 values in cardiac amyloidosis, suggesting myocardial edema in patients with CA. Ridouani et al. found that the myocardial native T2 values were significantly higher (*p* < 0.001) in patients with CA than in healthy subjects [5]. In our series, we confirmed this result, which strengthens the hypothesis of myocardial edema in CA patients. Furthermore, Ridouani et al. found significantly (*p* < 0.0001) higher myocardial native T2 values in AL-CA (63.2 ± 4.7 ms) than in ATTR-CA (56.2 ± 3.1 ms) patients. We also found a more pronounced native T2 relaxation time in AL-CA compared to ATTR-CA patients, even if we did not observe statistically significant differences, probably due to the non-uniform study population, with a scant subset of AL-CA patients.

Recently, Fontana et al. reported higher myocardial T1 and lower ECV values in AL-CA than in ATTR-CA patients [17]. In our series, we found an elevation in native T1 values in both types of amyloidosis without statistically significant differences, while, in line with Fontana et al., we reported less pronounced ECV values in AL-CA compared to ATTR-CA patients. Nevertheless, we did not detect a significant difference in the ECV between the two types of amyloidosis. However, even our results suggest that the elevation of myocardial T1 values in CA patients is due not only to amyloid infiltration but also to myocardial edema. Therefore, native T1 values should be analyzed considering ECV values, which represent the purest measure of amyloid burden. Furthermore, in light of these results, we can also deduce that a comprehensive evaluation of cardiac amyloidosis incorporating not only myocardial T1 but also myocardial T2 imaging is necessary.

Interestingly, the study by Kotecha et al. demonstrated differences between the native T2 values of treated and untreated AL amyloidosis patients, suggesting that myocardial T2 imaging could be used to monitor the response to therapy in cardiac amyloidosis [18]. This result is an important insight for evaluating the possible prognostic value of T2 mapping, and further work is needed.

The present study has some limitations. First, this was a retrospective observational study. Second, the number of subjects included in this work was relatively small because it was a single-center study. Finally, the patient cohort was non-uniform, with a low number of AL-CA patients, which could explain why we did not observe statistically significant differences between the native T2 values of the AL-CA and ATTR-CA patients. Despite these restrictions, our study emphasizes the high level of myocardial T2 value elevation in CA patients. Another limitation is that running an extra sequence is obviously time-consuming. However, adding T2 mapping to the CMR protocol for CA evaluation only lengthens the execution time by a few minutes. Even the segmentation process for T2 mapping does not take long if the radiologist is experienced and uses automatic software.

## 5. Conclusions

In this study, we demonstrated a significant increase in myocardial native T2 values in cardiac amyloidosis. T2 value elevation was observed in both types of amyloidosis. These findings support the concept of the presence of myocardial edema in cardiac amyloidosis. Therefore, CA is now emerging as a disease that is not purely infiltrative. This study highlights the additive value of T2 mapping in the evaluation of cardiac amyloidosis, allowing for more comprehensive tissue characterization. The presence of alterations in T2 maps could be useful for evaluating the stage of the disease and perhaps for optimizing therapies. CMR protocols restricted to myocardial T1 mapping carry the risk of missing myocardial edema, which could play a role in myocardial injury.

## Figures and Tables

**Table 1:** Scanner reference ranges assessed in our center.

Scanner Reference Range	Magnetom Sola	Magnetom Aera	Philips Ingenia
Native T1	950–1050 (1000 ± 50)	969–985 (977 ± 8)	974–1014 (994 ± 20)
Native T2	45–48 (46.5 ± 1.5)	49–52 (50.5 ± 1.5)	54–55 (54.5 ± 0.5)
ECV	25.3–26.3 (25.8 ± 0.5)	25.3–26.3 (25.8 ± 0.5)	24.7–26.6 (25.6 ± 0.9)

**Table 2:** Overview of CMR scan parameters according to scanner type. FOV = field of view; TR = repetition time; TE = echo time; FA = flip angle; ST = slice thickness; IT = inversion time.

		SA-Cine	SA-Native T1 Mapping	SA-LGE	SA-T2 TSE	SA-Native T2 Mapping
FOV	1	300	300	300	300	300
	2	300	300	300	300	300
	3	300	300	300	300	300
TR (ms)	1	30.8	305.8	789	1666.2	218.2
	2	3.2	2.2	4.5	1739.1	869.6
	3	30.8	278.5	676	1459.3	218.2
TE (ms)	1	1.3	1.1	1.1	60	1.0
	2	1.6	0.99	2.1	70	8.4
	3	1.3	1.0	1.1	60	1.0
FA (°)	1	60°	35°	40°	180°	70°
	2	60°	35°	20°	90°	90°
	3	60°	35°	40°	180	70°
ST (mm)	1	8	8	8	8	8
	2	8	10	8	8	10
	3	8	8	8	8	8
IT (ms)	1		109	285		
	2		350	300–380		
	3		100	285		

(1) MAGNETOM Aera, Siemens Healthineers, Erlangen, Germany; (2) Philips Ingenia, Philips, Best, the Netherlands; (3) MAGNETOM Sola, Siemens Healthineers, Erlangen, Germany.

**Table 3:** Clinical and echocardiographic population characteristics.

Characteristics	ATTR-CA (n = 51, 73%)	AL-CA (n = 19, 27%)	*p*-Value
Clinical			
Age	77 ± 8	71 ± 7	*p=* 0.01
Male	43 (84%)	9 (47%)	*p=* 0.02
BSA	1.89 ± 0.2	1.72 ± 0.2	*p=* 0.03
NYHA (I, II, III, IV)	I/II 92%	I/II 84%	*p=* 0.50
NT-pro BNP (pg/mL)	2450 ± 2711	2386 ± 1870	*p* = 0.93
eGFR (mL/min)	62 ± 27	70 ± 16	*p=* 0.15
Echocardiography			
Transmitral E/A	1.36 ± 0.9	1.28 ± 0.7	*p=* 0.72
E/E’	15.7 ± 6.3	13.7 ± 5.2	*p=* 0.92

**Table 4:** MRI data.

MRI Parameters	ATTR-CA (n = 51, 73%)	AL-CA (n = 19, 27%)	*p*-Value
LV-EF (%)	50 ± 12	62 ± 14	*p* = 0.003
LV-SV (ml)	76 ± 20	66 ± 13	*p* = 0.018
LV-EDV (ml)	157 ± 41	111 ± 31	*p* < 0.001
LV-ESV (ml)	81 ± 34	45 ± 27	*p* < 0.001
LV-mass (indexed) g/m^2^	109.5 ± 37.8	78.9 ± 26.2	*p* < 0.001
IVS (mm)	18 ± 4	15 ± 3	*p* = 0.003
LGE	48 (96%)	19 (100%)	*p* = 0.376
QALE score	10.8 ± 4.0	8.5 ± 5.3	*p* = 0.049
Atrial LGE	37 (74%)	10 (53%)	*p* = 0.089
Native T2 (ms)	53.2 ± 4.5	56.0 ± 5.5	*p* = 0.260
Native T1 (ms)	1066 ± 59	1062 ± 42	*p* = 0.774
ECV	49 ± 10	44 ± 12	*p* = 0.202

## Data Availability

The datasets presented in this article is unavailable due to privacy. Requests to access the datasets should be directed to giulia.grazzini@unifi.it.
